# Case of a 57‐year‐old woman with acute confusion and inability to recognize her husband

**DOI:** 10.1002/acn3.52289

**Published:** 2025-01-07

**Authors:** Elizabeth Anderson

**Affiliations:** ^1^ Barrow Neurological Institute Phoenix Arizona USA; ^2^ St. Joseph's Hospital and Medical Center Phoenix Arizona USA

## Summary of Case

**Figure 1:**
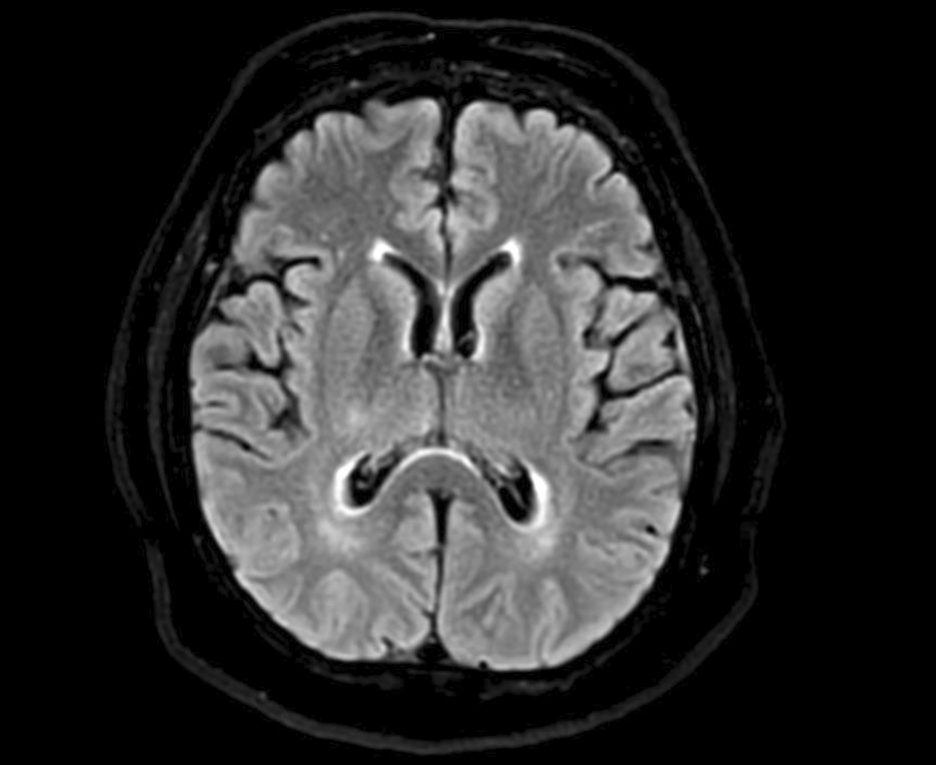
MRI brain w/wout contrast. Notable for chronic lateral right thalamic infarct, mild generalized volume loss. No acute intracranial abnormality. Ambra Imaging, Dignity Health Systems/St. Joseph Hospital Medical Center.

Patient is a 57‐year‐old Hispanic female with a past medical history of hypertension, asthma, and type II diabetes mellitus who presented to the emergency room (ER) for evaluation of 7 days of altered mental status. Upon initial presentation, the patient had difficulty following one step commands and was not fully oriented. Additionally, she appeared to be responding to external auditory and visual stimuli. Magnetic resonance imaging (MRI) completed in the ER was without acute intracranial abnormality (Figure 1), and electroencephalogram (EEG) was significant for diffuse slowing but no epileptiform activity was noted. She was admitted to the neurology service for further workup and treatment for possible autoimmune encephalitis, plasma exchange (PLEX), high‐dose steroids, as well as intravenous immunoglobulin (IVIG) treatments. However, despite treatments, the patient continued to remain altered. Therefore, psychiatry was consulted as well as a cognitive specialist. Per patient's husband, he was able to recall that she had in fact had several years of cognitive decline, periods of altered mental status, hallucinations, and slowed gait. During her admission she had periods of not recognizing her husband, and identified him as an imposter, consistent with Capgras syndrome.^1^ Given clinical findings, our cognitive team determined the patient's most likely diagnosis and started her on rivastigmine 1.5 mg bid with improvement.^2^

## Diagnosis

Lewy body dementia.

## Take‐Home Points

- It is important to elicit a thorough history from family members and other collateral, including medications, comorbidities, and duration of symptoms when patients present with change in mental status.
- Lewy body dementia can be difficult to recognize clinically, as it is often insidious in onset and can present similarly to other conditions. However, to make a diagnosis of probable dementia with Lewy bodies, in addition to dementia, two additional features must be present, which include cognitive fluctuations, visual hallucinations, REM sleep behavior disorder, and parkinsonism.^3^
- To aid in diagnosis, one can utilize the revised criteria for the clinical diagnosis of probable and possible dementia with Lewy bodies (DLB), which is set of clinical features and biomarkers used to identify if cognitive decline is secondary to Lewy body pathology (Table 1).
- Once recognized clinically, treatment of Lewy body dementia is supportive, and includes cholinesterase inhibitors, memantine, and levodopa. New‐generation medications are also being introduced to help with psychosis/hallucinations. However, there are currently no preventative treatments for this disease process.^2^

**Table 1:** Revised criteria for the clinical diagnosis of probable and possible dementia with Lewy bodies (DLB).

Essential feature	Dementia; progressive cognitive decline of sufficient enough magnitude to interfere with normal social/occupational functions
Core clinical features	Fluctuating cognition (variations in attention/alertness) Recurrent visual hallucinations (well formed/detailed) REM sleep behavior disorder (can precede cognitive decline)
Supportive clinical features	Severe sensitivity to anti‐psychotic medications Postural instability, repeated falls, syncope or unresponsiveness Severe autonomic dysfunction Hypersomnia or hyposomnia Hallucinations (in other modalities than mentioned) Systematized delusions Apathy, anxiety, or depression
Indicative biomarkers	Reduced dopamine transporter uptake on SPECT or PET Abnormal myocardial scintigraphy Polysomnographic confirmation of REM sleep disorder without atonia
Supportive biomarkers	Relative preservation of medial temporal lobe structures on CT or MRI Generalized low uptake on SPECT or PET perfusion/metabolism scan Prominent posterior slow‐wave activity on EEG with periodic fluctuations in the pre‐alpha/theta range
*Diagnosis*	
Probable DLB	
Two or more core clinical features of DLB are present, with or without the presence of indicative biomarkers, or Only one core clinical feature is present, but with one or more indicative biomarkers	
Possible DLB	
Only one core clinical feature of DLB is present, with no indicative biomarker evidence, or One or more indicative biomarkers is present but there are no core clinical features	

*Source*: McKeith, I. G., Boeve, B. F., Dickson, D. W., Halliday G., Taylor, J. P., & Kosaka, K. Diagnosis and management of dementia with Lewy bodies. Fourth consensus report of the DLB Consortium. *Neurology* Jul 2017, 89 (1) 88‐100; DOI: 10.1212/WNL.0000000000004058.
